# Behavioural Change Interventions for Preventing Periodontal Disease in Older Adults: A Literature Review

**DOI:** 10.3390/geriatrics10040097

**Published:** 2025-07-22

**Authors:** Stephanie Chu, Chun Hung Chu, Alice Kit Ying Chan

**Affiliations:** Faculty of Dentistry, The University of Hong Kong, Hong Kong 999077, Chinachchu@hku.hk (C.H.C.)

**Keywords:** older adult, elderly, oral health, prevention, periodontal, plaque control, behavioural change

## Abstract

Periodontal disease is a common and serious oral disease among older adults. As the global older population increases, preventing periodontal disease is vital for healthy ageing. Poor oral hygiene, uncontrolled diabetes, and smoking are key risk factors of periodontal disease. Improving oral hygiene, diabetes management, and quitting smoking are essential health behavioural change interventions to manage periodontal disease. The objective of this study is to review the prevention of periodontal disease among older adults through health behavioural change interventions. Effective strategies to improve oral hygiene include personalised education on proper brushing and interdental cleaning. Educating caregivers is equally important as they supervise care-dependent older adults to maintain oral health. For those with diabetes, physical activity improves glycated haemoglobin levels and clinical periodontal parameters by reducing reactive oxygen species and systemic inflammation. Smoking cessation could be achieved through a multi-faceted approach. Effective smoking cessation combines brief interventions with intensive behavioural/pharmacological support for long-term success, especially in highly dependent individuals. Tailored strategies for older adults, integrated care, and expanded research improve outcomes and health equity in ageing populations. In conclusion, health behavioural change interventions are non-invasive preventive measures that include oral hygiene reinforcement, diabetic management, and smoking cessation. Prioritising these interventions empowers older adults to maintain oral health, reducing disease burden and enhancing overall well-being for healthy ageing.

## 1. Introduction

The global population of older adults, defined by the World Health Organization (WHO) as individuals aged 65 and above, is expanding at an unprecedented rate [1]. By 2050, it is projected that one in six people worldwide will belong to this demographic, totalling 1.6 billion individuals, with nearly 460 million aged 80 or older [2]. As of 2024, global life expectancy averages 73.3 years and is expected to rise [3]. Although population ageing reflects social, economic, and medical advancements, increased lifespans also mean prolonged exposure to chronic health conditions, including oral diseases [4]. Among these conditions, periodontal disease is a pervasive, severe non-communicable disease affecting over 60% of older adults [1]. It is characterised by dysbiotic plaque biofilm and the dysregulation of host immune response, resulting in soft tissue destruction and alveolar bone loss [5]. This chronic inflammatory condition not only impairs oral function but exacerbates systemic morbidities including cardiovascular disease, diabetes, and cognitive decline [6,7,8]. Given the extensive repercussions of periodontal disease, prevention through cost-effective behavioural interventions has emerged as a critical strategy to promote healthy ageing [4]. The objective of this study is to review the prevention of periodontal disease among older adults through health behavioural change interventions.

### 1.1. Oral Hygiene and Periodontal Disease

Poor oral hygiene, an established modifiable risk factor, allows for bacterial plaque build-up to trigger gingival inflammation [9]. Periodontal disease arises from a complex interplay between plaque biofilm and host inflammatory responses [10]. Age-related challenges, including immune senescence, frailty, systemic comorbidities, and cognitive decline, heighten vulnerability to disease progression [11]. For care-dependent older adults, maintenance of oral hygiene presents additional challenges of reduced mobility, heightened gag reflexes, or resistance to assisted care [12]. Studies reveal that poor oral health in hospitalised geriatric patients correlates with higher mortality rates, underscoring detrimental consequences to neglected periodontal care [13].

### 1.2. Systemic Health Conditions and Periodontal Disease

Beyond oral hygiene, systemic health significantly influences periodontal outcomes [14]. Epidemiological studies confirm that diabetes and smoking are major risk factors of periodontitis [7,15]. Diabetes mellitus, the most common endocrine disorder amongst the geriatric population, increases the incidence and progression of periodontitis by 86% [1]. Over half of older adults with diabetes develop periodontitis as an oral complication [16]. Hyperglycaemia dysregulates immune defences, intensifies periodontal destruction, and undermines wound healing [17]. Conversely, periodontitis dysregulates glucose homeostasis and has been shown to increase mortality rates in subjects with type 2 diabetes, thus sharing a bidirectional relationship [7,18]. In addition, smoking adversely affects systemic health [19]. It is a powerful risk indicator for periodontal attachment loss and hastens periodontal destruction by impairing blood flow, suppressing immune responses, and promoting the growth of periodontal pathogens [20,21]. Risks for severe attachment loss intensify with age and smoking duration [22,23].

### 1.3. Prevention of Periodontal Disease

Preventive strategies focusing on health behavioural changes offer a cost-effective approach to mitigate periodontal disease [24]. Oral hygiene education tailored to older adults and their caretakers is well recognised as a fundamental step for disease prevention [25]. Mechanical and chemical removal of plaque by self-performed oral hygiene practices can significantly reduce gingival inflammation, a key risk factor for the onset of periodontitis [15]. For diabetic patients, integrating regular physical activity and healthy dietary regimens to improve glycaemic control indirectly reduces the severity and prevalence of periodontal disease [18]. Similarly, smoking cessation programmes demonstrate measurable benefits in slowing disease progression and enhancing treatment outcomes [26].

The European Federation of Periodontology advocates the aforementioned lifestyle modifications as pillars of periodontal prevention [27]. Evidence supports these strategies in improving periodontal parameters and oral health-related quality of life [18,28,29,30] in older adults with declines in cognitive, medical, and physical function. Behavioural change intervention applied to other age groups may not be able to be applied in older adults [12]. However, research on behavioural change intervention specific to older adults remains sparse, leaving gaps in understanding ways to adopt behavioural interventions tailored to the unique needs of the geriatric population. Socioeconomic disparities, limited healthcare access, physical or cognitive disabilities, and low health literacy may further complicate implementation, necessitating cost-effective public health measures to reinforce preventive interventions [31].

**Figure 1:**
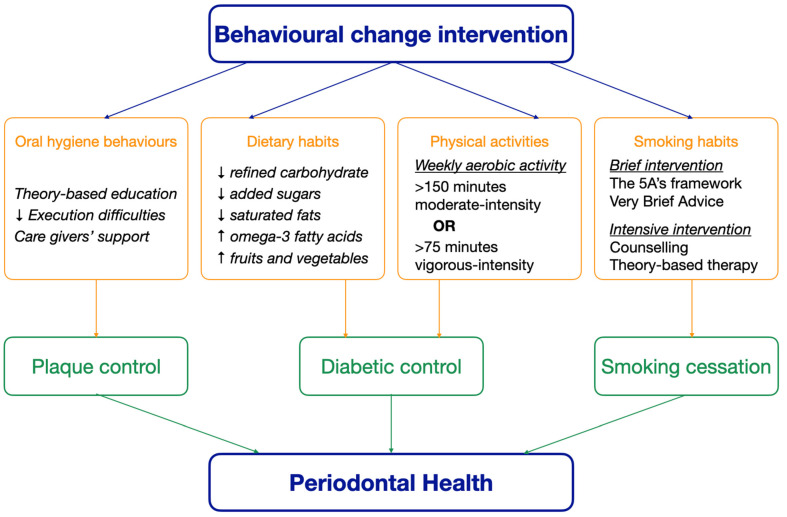
The effect of behavioural change interventions on periodontal health.

Oral hygiene improvement, diabetes management, and smoking cessation are essential health behavioural change interventions for the management of periodontal disease [18,28,29,30]. Collaborative efforts among healthcare providers, caregivers, and policymakers are essential to ensure equitable access to these interventions [25]. Empowering older adults through preventive measures not only preserves oral health but defines healthy ageing by enhancing overall well-being and functional ability [32]. With the global trend of the population ageing, prioritizing such initiatives becomes imperative. The aim of this literature review is to review the prevention of periodontal disease among older adults through health behavioural change interventions, including oral hygiene improvement, diabetic control, and smoking cessation. Figure 1 shows how behavioural change interventions maintain periodontal health in older adults.

This review included clinical studies investigating the effects of behavioural change interventions in controlling the three periodontal risk factors, plaque accumulation, diabetic condition, and smoking habit, with any participants aged 65 or above. The keywords included “plaque control”, “diabetic control”, “smoking cessation”, “behavioural change”, and “periodontal health”. The research question was whether behavioural change interventions in controlling periodontal risk factors were effective in preventing periodontal diseases in older adults. Table 1 lists the clinical studies investigating the effects of behavioural change interventions on the periodontal health of older adults.

## 2. Oral Hygiene Maintenance Through Education and Practice

Behavioural interventions to maintain oral cleanliness require a dual approach of targeted oral health education and the adoption of daily hygiene practices tailored to the unique challenges of ageing. Plaque control is the cornerstone of preventing periodontal disease and essential for all methods of periodontal therapy [31].

### 2.1. Oral Health Education

Effective oral health education lies in inspiring motivation for change and treatment compliance [24]. While standardised, lecture-based oral health education is often implemented in public health interventions, such approaches may fail to account for individual age-related barriers including cognitive decline, reduced dexterity, or limitations in self-efficacy. Instead, personalised interventions guided by behavioural psychology and theoretical frameworks have proven impactful among older adults [31]. For example, interventional studies testing the health belief model and social cognitive theory are emerging and have shown improvements to oral health perception, knowledge, behaviour, self-efficacy, and oral health-related quality of life among older adults. Motivational interviewing, a form of cognitive behavioural therapy that fosters intrinsic motivation for change, has emerged as a cost-effective tool for successful oral health education [24,43]. Schensul and coworkers tested this model against community-based campaigns among older adults in senior residences, combining motivational interviewing with visual demonstrations, practical training, and collaborative goal-setting. In addition to decreased plaque and gingival indices, improvements to emotional and behavioural modifying factors were observed, including reduced fears towards oral diseases, stronger intentions to maintain oral cleanliness, and greater self-efficacy towards hygienic routines [36]. Similarly, a study in the United States found that individual-based motivational interviewing among older adults significantly correlated with improved scores in self-efficacy and oral health-related quality of life [44]. The integration of the 5S (Sort, Set in order, Shine, Standardise, and Sustain) methodology in oral hygiene interventions to improve plaque control has been proposed for older adults with cognitive decline. This model aims to build up long-term oral hygiene behaviour changes by repeatedly performing self-regulated, standardized oral hygiene practices and removing execution difficulties among geriatric patients. However, its clinical effectiveness among older adults requires further investigation [45]. These findings underscore the importance of addressing cognitive, emotional, and behavioural factors to inspire change, which directly affects compliance and the success of oral hygiene interventions.

Personalised training programmes further enhance outcomes by adapting to individual needs. Hospitalised older adults taught to brush and clean their dentures using personal tools, supplemented with adaptive aids such as silicone grips for weak grip and glasses for impaired visual acuity, demonstrated marked improvements in plaque removal and autonomous oral hygiene practice [38]. Furthermore, repetition and reinforcement are equally critical. Axelsson and colleagues performed a 30-year plaque control programme pairing repeated education with mechanical plaque removal. The results revealed limited tooth mortality and attachment loss with a mean increase of 0.3–0.4 millimetres in probing attachment levels, thus reflecting the importance of education reinforcement for successful treatment outcomes [46].

Among care-dependent older adults, caregiver education is indispensable. This demographic may present with reduced mobility, sensitivity to gag reflexes, or cognitive impairments that complicate assisted oral hygiene [47]. A systematic review evaluating targeted oral health interventions for older adults found more reports of significant plaque reduction among studies that incorporated caregiver education than those that only involved older adults [31]. However, gaps remain in understanding the long-term periodontal implications of health educational programmes, calling for further research to explore the impact of caregiver education on the oral health of care-dependent populations [48].

### 2.2. Oral Hygiene Practices

The mechanical removal of plaque biofilm is fundamental for the maintenance of oral hygiene. Achieving oral cleanliness requires tools and techniques adapted to age-associated physical and cognitive limitations common to older adults. Toothbrushing at least twice daily is well supported as an essential practice, with guidance recommended to ensure adequate techniques, particularly on tooth surfaces with difficult access [49,50]. The Bass method, which involves angling bristles at 45 degrees to the gumline, has proven effective in reducing plaque retention [33]. There is heterogeneity between studies comparing the effectiveness of powered versus manual toothbrushes, suggesting that both can achieve comparable results when used with proper technique [35,51,52,53,54,55]. However, there is considerable evidence supporting greater efficacies in plaque removal and controlling gingivitis from the use of powered toothbrushes [37,52,56]. Furthermore, powered toothbrushes have been recommended for ease of use among older adults who experience physical or cognitive limitations including frailty, frequent gag reflexes, deteriorating vision, reduced manual dexterity, or arthritis [57].

Toothbrushing alone removes only 60% of dental plaque, necessitating interdental cleaning in daily practice [58]. Higher risks of periodontitis are associated with the lack of interdental cleaning by dental floss or interdental brushes [59]. Dental floss, although effective in reducing gingival inflammation and periodontitis, poses challenges for older adults with limited dexterity [34]. Technical difficulties of usage and risk of soft tissue trauma have led to growing preference for interdental brushes [60]. Recommended by the European Federation of Periodontology for wider embrasure spaces, these brushes mechanically remove plaque up to 2.5 millimetres below the gingival margin and have been deemed the most effective method for interproximal cleaning [58,61]. In comparative studies, interdental brushes outperformed floss in reducing plaque, gingival bleeding, and pocket depths, with higher patient acceptance [62,63]. Despite this, a Cochrane review cautions that evidence to support significant differences is limited, and further well-designed clinical trials are warranted [64].

Chemical plaque control has been investigated as an adjunct and, in limited cases, a replacement to mechanical methods where physical and cognitive decline is severe [65]. Strong evidence distinguish chlorhexidine as the gold standard for chemical plaque removal due to its substantivity and marked efficacy in reducing plaque and gingival inflammation [66,67,68,69]. However, repercussions from the long-term use of chlorhexidine including extrinsic tooth staining, burning sensations, disturbed taste, and oral mucosal irritation call for herbal alternatives, particularly for older adults who cannot achieve effective plaque control by mechanical removal alone [70]. Among herbal formulations, propolis mouthrinses have shown comparable effects in plaque and bleeding reduction when compared with chlorhexidine, with one study reporting superior outcomes in improving periodontal parameters [71,72]. Propolis exerts antioxidant, antimicrobial, anti-inflammatory, and immunomodulatory properties, providing promising potential in clinical applications [71]. Further studies are recommended to support its therapeutic role as a safe and viable alternative to chlorhexidine in managing periodontal disease.

## 3. Diabetic Control Through Physical Activity and Dietary Management

The management of diabetes mellitus, a chronic disorder characterised by hyperglycaemia, relies heavily on lifestyle modifications [40]. Lifestyle modifications include increased physical activity and the adoption of energy-restricted diets [73]. These have been shown to induce better performance than pharmacologic means alone in preventing diabetes, reducing its risk by 58% in people with impaired glucose tolerance [73,74].

Combining lifestyle modifications with dental care enhances the success of diabetic and periodontal therapy. A randomised controlled trial to study periodontal status among older adults with diabetes tested the efficacy of repeated lifestyle counselling with oral hygiene education, emphasizing healthy dietary habits, physical activity, weight loss, and smoking cessation. The results after six months showed significant reductions in glycated haemoglobin, fasting plasma glucose, plaque and gingival indices, probing depths, and attachment loss [40]. Similarly, a six-month intensive diabetic care programme combining exercise, dietary adjustments, and patient education demonstrated significant improvements in glycated haemoglobin (HbA1c), fasting glucose, and periodontal parameters, independent of calculus removal. These findings confirm that the comprehensive control of diabetes alone can significantly reduce hyperglycaemia-induced periodontal inflammation, although the removal of subgingival calculus must also be received to eradicate pathological periodontal pockets and holistically restore periodontal health [75].

### 3.1. Physical Activity

Physical activity, defined by the WHO as any bodily movement requiring energy expenditure, is key for diabetes management [76]. For older adults, the WHO recommends at least 150 min of moderate-intensity aerobic activity or 75 min of vigorous-intensity exercise weekly [77]. Such regimens enhance glycaemic control by reducing HbA1c levels and improving insulin sensitivity. A randomised controlled trial conducted among patients with type 2 diabetes found that regular aerobic exercise and resistance training resulted in improvements to both HbA1c levels and clinical periodontal parameters within six months [41]. A study among Japanese subjects found that low body mass index and high maximal oxygen consumption from physical activity were inversely associated with severe periodontitis, suggesting that obesity and low physical fitness increased the risk of periodontal disease [78]. The inverse, linear relationship between physical activity and the risk for periodontitis is further supported in a prospective study, which reported greater average bone loss among those who were less physically active [79].

Regular physical activity reduces systemic levels of reactive oxygen species, harmful molecules that exacerbate inflammation and tissue damage [41]. In diabetic patients, elevated levels of reaction oxygen species contribute to both insulin resistance and periodontal degradation due to shared inflammatory pathways [80]. Studies show that physically active individuals with type 2 diabetes exhibit lower concentrations of reactive oxygen species, correlating with improved lipid profiles, blood pressure, and cardiovascular outcomes. Furthermore, such patients demonstrate better periodontal health, including reduced plaque accumulation, gingival inflammation, and clinical attachment loss [81].

Physical activity modulates inflammatory biomarkers central to the pathogenesis of diabetes and periodontitis. Chronic hyperglycaemia elevates levels of pro-inflammatory cytokines such as C-reactive protein and interleukin-1β, which impair immune function and accelerate periodontal destruction. Regular exercise downregulates cytokine production and thereby reverses systemic inflammation [82]. In fact, a lack of physical activity for as few as 30 days has been shown to reverse cytokine levels back to their initial concentrations, thus reflecting the imperative role of physical activity in immune regulation for patients with type 2 diabetes.

### 3.2. Dietary Control

Dietary interventions are equally vital in diabetes management, with nutrition serving as a modifiable determinant of both glycaemic control and periodontal health [29]. Carbohydrate regulation is central for diabetes prevention. Refined carbohydrates and added sugars aggravate oxidative stress, insulin resistance, and hyperglycaemia. In addition, increased risk and prevalence for periodontal disease occur due to triggering of hyper-inflammatory mechanisms, microbial dysbiosis, and the apoptosis of periodontal ligament cells [83,84,85]. Conversely, minimally processed, fibre-rich carbohydrates stabilise blood glucose and reduce the risks of diabetes, periodontitis, and other non-communicable diseases [86]. An inverse relationship exists between dietary fibre intake and periodontal disease [87]. In an interventional study, patients with type 2 diabetes complied to a 12-week low-carbohydrate diet and demonstrated reduced periodontal probing depths and serum concentrations of C-reactive protein and interleukin-6, thereby illustrating the protective effects of dietary fibre against inflammatory diseases [39].

The effects of dietary fats on diabetes and periodontal disease vary based on the nature of fatty acids consumed. Diets high in saturated fatty acids are discouraged in diabetic therapy due to impairment of glucose metabolism [88]. The associated production of low-density lipoprotein cholesterol activates pro-inflammatory cascades and increases susceptibility to hyperlipidaemia and reduced insulin sensitivity. Greater risks for periodontitis also occur due to heightened oxidative stress and the associated increase in the intensity and duration of inflammatory processes [89,90]. While omega-3 polyunsaturated fatty acids exert protective effects, an imbalance in omega-6 to omega-3 ratio exacerbates inflammation, as seen in patients with greater periodontal bone loss [91,92,93]. Omega-3 fatty acids, including eicosapentaenoic acid and docosahexaenoic acid, inhibit prostaglandin synthesis and periodontal pathogens, thus reversing gingival inflammation and reducing alveolar bone loss [93]. Studies emphasise the importance of prioritizing omega-3 rich foods to counteract the pro-inflammatory repercussions of omega-6 heavy diets. In addition, anti-inflammatory dietary patterns, such as the Mediterranean and Dietary Approaches to Stop Hypertension (DASH) diets, have been shown to be effective in combating diabetes and periodontitis [94,95]. Rich in whole grains, fruits, vegetables, and healthy fats, these regimens lower HbA1c, fasting glucose, and body weight. Extra-virgin olive oil, a Mediterranean diet staple, reduces the risk of diabetes through its anti-inflammatory and antioxidant properties HbA1c [29].

## 4. Smoking Cessation Through a Multi-Faceted Approach

Tobacco smoking is a key modifiable risk factor for periodontal disease, with studies consistently showing that former smokers experience significantly better periodontal health from reduced plaque, shallower probing depths, and less tissue destruction compared with current smokers [42,96,97,98]. Smoking cessation has been shown to significantly reduce the risk for severe periodontitis in a time-dependent manner [98]. Costa and coworkers reported that former smokers had a 1.8-fold risk of advanced periodontitis compared with never-smokers, far lower than the 4.8-fold risk observed in current smokers [42]. The U.S. national health surveys in 2009–2012 in the United States estimated a 4% decrease in risk per year of cessation, with Torrungruang and coworkers reporting that risk levels return to those of never-smokers after at least 10 years depending on smoking exposure [98,99]. Similarly, the risk for periodontitis-associated tooth loss was found to reduce by 6% per year of cessation, and a maintenance period of 15–20 years could revert risk levels back to those of non-smokers [100,101]. Evidence of this time-dependent relationship may guide periodontal maintenance and recall intervals as patients progress in age.

The addictive nature of nicotine necessitates sustained, patient-centred strategies to inspire motivation and behavioural change. Research underscores that smokers receiving guided cessation support from healthcare professionals exhibit significantly higher odds of long-term success and lower failure rates compared with those without structured interventions [102]. Cessation programmes often require a multi-faceted approach tailored to the patient’s individual perceptions and needs.

### 4.1. Brief Interventions: Efficiency and Limitations

Brief interventions are evidence-based approaches that have been shown to increase quit rates in a time-effective and cost-efficient manner [103]. The 5A’s framework (Ask, Advise, Assess, Assist, Arrange) and Very Brief Advice (VBA) protocol, which can be delivered in as little as three minutes, have proven effective in initiating cessation attempts [103]. These strategies prioritise simplicity, enabling healthcare providers to promptly identify smoking habits, deliver personalised advice, and connect patients to resources. Remote interventions, such as telephone counselling and short message service-based support, can further enhance accessibility, particularly for immobile older adults or those with limited access to in-person dental care [104]. However, while brief interventions are cost-effective and well received, their efficacy in sustaining long-term abstinence remains debated. Some studies highlight their limited impact on preventing relapse, suggesting that individuals with heavy nicotine dependence may require more intensive, tailored support to achieve lasting behavioural change [105].

### 4.2. Intensive Interventions: Combining Behavioural and Pharmacological Therapies

For smokers with a high nicotine dependence or long history of smoking, intensive interventions may yield superior outcomes [106]. A Cochrane review analysing 15 trials found that intensive behavioural therapy provided a small but statistically significant advantage over minimal advice, underscoring the value of structured, repeated engagement [103]. Counselling and cognitive-behavioural therapy address maladaptive thought patterns and reinforce self-efficacy, correlating with successful cessation outcomes. Motivational interviewing, a collaborative counselling approach to strengthen personal motivation, has demonstrated efficacy towards sustained abstinence, particularly when integrated with other interventions [107]. Combining behavioural interventions with pharmacotherapy such as nicotine replacement therapy, varenicline, or bupropion further enhances success rates by alleviating withdrawal symptoms [105]. For example, a longitudinal trial integrating counselling, nicotine replacement therapy, and periodontal treatment among middle-aged smokers reported that 12-month quit rates compared favourably with those achieved in specialist clinics, demonstrating the potential of multi-faceted, dental-based cessation programmes [108]. The success of such integrated programmes underscores the ethical imperative for oral health practitioners to adopt proactive roles in smoking cessation.

### 4.3. Tailored Strategies for Older Adults

Notably, older adults present unique challenges and may require tailored approaches due to higher rates of comorbidities linked to higher tobacco dependence, such as depression or schizophrenia [109]. At present, similar studies conducted among older adults are lacking and are required to further verify the efficacies of such cessation techniques. Tailored approaches must account for these factors, integrating mental health support and addressing age-specific barriers like mobility limitations or social isolation. Remote interventions and collaborative care models involving dentists, physicians, and psychologists are particularly relevant for this demographic. Notably, older adults are underrepresented in smoking cessation research, creating gaps in evidence-based protocols for this population. Future studies must prioritise this group to validate strategies that balance efficacy with practicality. Expanding research on older populations and refining integrated care models will further strengthen cessation outcomes, advancing health equality in a global ageing population.

## 5. Conclusions

Periodontal disease is a widespread and serious phenomenon among older adults globally. Oral hygiene improvement, diabetes control, and smoking cessation are key behavioural changes for the prevention of periodontal disease. Prioritizing these strategies enhances oral health, reduces health burdens, and supports healthier ageing. This review demonstrates how these interventions can enhance quality of life and long-term well-being in ageing populations. More well-designed randomized clinical trials should be conducted to provide evidence for their uses in geriatric care.

## Figures and Tables

**Table 1:** Clinical studies of behavioural change interventions on older adults’s periodontal health

Study Type (Authors, Year)	Design (Participants, Study Period, Grouping)	Findings
***Plaque control***		
Randomized clinical trial [33]	30 aged ≥65 6 months Group 1: Bass brushing method Group 2: Usual care	The Bass brushing method was a simple and effective oral hygiene practice that reduced dental plaque in older adults hospitalized with pneumonia after discharge.
Longitudinal study [34]	685 aged ≥65 5 years Group 1: Flossing Group 2: No flossing	Flossing was an important oral hygiene behaviour to prevent oral disease progression in older adults
Randomized clinical trial [35]	60 aged ≥60 2 weeks Group 1: Electric toothbrush Group 2: Soft toothbrush	Conventional and electric toothbrushes were effective in removing bacterial plaque within the elderly group. No significant differences were detected.
Randomized clinical trial [36]	332 aged 50–90 1 month Group 1: The face-to-face counselling Group 2: The oral health campaign	A face-to-face counselling intervention improved gingival index and plaque score outcomes in older adults.
Randomized clinical trial [37]	14 aged 68–85 6 months Group 1: Powered toothbrush Group 2: Manual toothbrush	The powered toothbrush was more effective than a regular manual toothbrush in removing plaque and controlling gingivitis.
Randomized clinical trial [38]	90 adults aged ≥60 12.4 ± 3.6 days Group 1: Individual oral health care training Group 2: No training	Individual oral health care training improved oral and denture hygiene in geriatric inpatients.
***Diabetic control***		
Randomized clinical trial [39]	100 aged 45–68 12 weeks Group 1: Low-carbohydrate diet Group 2: Regular dietary habits	The adoption of a low-carbohydrate diet for 12 weeks showed significant improvements in periodontal health and a reduction in inflammation in patients with type 2 diabetes.
Randomized clinical trial [40]	66 aged >60 years 6 months Group 1: Lifestyle and oral health education Group 2: Routine program	Older adults receiving a lifestyle change plus dental care program had significantly lower glycated haemoglobin, fasting plasma glucose, plaque index, gingival index, probing depth, and attachment loss than those in the control group.
Randomized clinical trial [41]	37 aged 46–73 6 months Group 1: Physical activity Group 2: No intervention	Physical activity over a period of 6 months improved both periodontal health and glycated haemoglobin concentrations in patients with type 2 diabetes mellitus.
***Smoking cessation***		
Longitudinal study [42]	59 aged ≤50 and 83 aged >50 6 years Group 1: Non-smokers Group 2: Former smokers Group 3: Current smokers	During 6 years of periodontal maintenance therapy, cumulative smoking exposure and shorter time since smoking cessation were significantly associated with the recurrence of periodontitis.
